# Nucleoside Analog-treated Chronic Hepatitis B Patients showed Reduced Expression of PECAM-1 Gene in Peripheral Blood Mononuclear Cells in Bangladesh

**DOI:** 10.5005/jp-journals-10018-1108

**Published:** 2014-07-28

**Authors:** Nusrat Sultana, Shahina Tabassum, Saif Ullah Munshi, Marufa Hossain, Akhter Imam

**Affiliations:** 1 Department of Virology, Dhaka Medical College, Dhaka, Bangladesh; 2 Department of Virology, Bangabandhu Sheikh Mujib Medical University, Dhaka, Bangladesh; 3 Department of Microbiology, ZH Sikder Women’s Medical College, Dhaka, Bangladesh; 4 Department of Dentistry, Kurigram Sadar Hospital, Kurigram, Bangladesh

**Keywords:** PECAM-1, CD31, Conventional RT-PCR, PBMC, NA therapy.

## Abstract

**Background and aim:**

Assessment of therapeutic response is important for monitoring the prognosis and to take decision for cessation of nucleoside analogues therapy in chronic hepatitis B patients. In addition to serum alanine aminotransferase (ALT), hepatitis B virus (HBV) deoxyribonucleic acid (DNA) load and HBeAg status, identification of molecular markers associated with host immune response would be essential to assess therapeutic response. In this regard the current study was performed with the aim to detect expression of platelet endothelial cell adhesion molecule (PECAM)-I gene in peripheral blood monocytes (PBMCs) of treated chronic hepatitis B patients and also to correlate expression of this gene with serum HBV DNA load and serum ALT levels.

**Materials and methods:**

The study analyzed 60 chronic hepatitis B (CHB) patients, including 30 untreated and 30 nucleoside analogs treated and 10 healthy controls. PECAM-1 gene expression/ transcripts were detected by conventional RT-PCR.

**Results:**

The expression PECAM-1 mRNA in the PBMCs of CHB patients was significantly higher in untreated (3.17 ± 0.75) than the treated patients (1.64 ± 0.29) (p < 0.01). Expression of PECAM-1 was positively correlated with serum ALT levels of both untreated (r = 0.580) and treated (r = 0.566) CHB patients. Moreover, in both untreated and treated groups, these gene expressions were positively correlated to serum HBV DNA load with the correlation coefficient r = 0.545 and r = 0.591 respectively.

**Conclusion:**

PECAM-1 may be used as a biomarker for assessment of inflammatory activity as well as therapeutic response in CHB patients.

**How to cite this article:** Sultana N, Tabassum S, Munshi SU, Hossain M, Imam A. Nucleoside Analog-treated Chronic Hepatitis B Patients showed Reduced Expression of PECAM-1 Gene in Peripheral Blood Mononuclear Cells in Bangladesh. Euroasian J Hepato-Gastroenterol 2014;4(2):87-91.

## INTRODUCTION

About 350 to 400 million people worldwide are chronically infected with hepatitis B virus (HBV)^1^ and chronic hepatitis B (CHB) can lead to cirrhosis, hepatocellular carcinoma (HCC) and death.^2^ Antiviral therapy is used in CHB patients to reduce the liver damage and progression of disease.^3^ Among the therapeutic options duration of treatment with nucleos(t)ide analog (NA) is indefinite in comparison to IFN-alpha which is finite. However, main concern with long-term treatment by nucleos(t)ide analogs is emergence of antiviral-resistant mutations resulting in limiting future treatment options.^4^ Therefore, assessment of treatment response following NAs therapy is a major part of CHB management. Parameters used to assess treatment response include normalization of serum ALT, decrease in serum HBV DNA level, loss of HBeAg with or without detection of anti-HBe and improvement in liver histology.^5^ However, the molecular mechanisms underlying viral clearance following therapy are poorly understood. Therefore, molecular markers may be able to improve prediction and assessment of treatment responses in CHB patients.

Platelet endothelial cell adhesion molecule-1 (PECAM-1) (CD 31) is expressed on platelets, monocytes, neutrophils, NK and CD8+ T cells constitutively. High expression is observed on continuous endothelial cells at cell-cell borders^6,7^ and weak expression on sinusoidal endothelial cells (SEC).^8-10^ PECAM-1 plays an accredited role in the inflammatory process and leukocyte-endothelial interaction, especially in transmigration of leukocytes through intercellular junctions. PECAM-1 has the potential to mediate both homophilic adhesion and heterophilic binding to other molecules.^11^ It is associated with cell survival and angiogenesis.^12,13^ In addition PECAM-1 influences activation and regulates trafficking of integrins. It also increases T lymphocyte ability to bind to integrin substrates.^12^

However, the exact role of adhesion molecule in viral hepatitis immune pathogenesis is still uncertain. To the best of our knowledge, there is very little data regarding PECAM-1 in CHB available and there is no data with respect to its relationship with other adhesion molecules and antiviral therapy.

The purpose of the study was to compare PECAM-1 gene expression in PBMCs of treated and untreated CHB patients and to correlate expression of this adhesion molecule with serum HBV DNA load and ALT levels.

## MATERIALS AND METHODS

### Patients and Samples

Three groups of subjects were included in the present study. The first group comprised of 30 untreated HBsAg-positive CHB patients for ≥6 months with elevated alanine aminotransferase levels (≥ 2 times the upper limit of normal). The second group comprised 30 nucleos(t)ide analogs treated chronic hepatitis B patients and the third group comprised 10 healthy controls with no previous history or current evidence of any liver disease, normal aminotransferase levels, normal liver on ultrasound, and negative for HBsAg, antiHBeAg, IgG anti-HBcAg, anti-HCV, IgM antihepatitis E virus, IgM antihepatitis A virus and antihuman immunodeficiency virus. All healthy controls had not received vaccination for HBV in the past and were negative for total anti-HBc. Exclusion criteria were regular alcohol consumption, diabetes, severe systemic illness, pregnancy, hepatocellular carcinoma, coinfection with human immunodeficiency virus or other hepatitis viruses or immunosuppressive therapy for other associated illness. After Institutional Review Board (IRB) approval, informed written consent was obtained from all cases.

About 5 ml of blood sample was collected from each patient by aseptic venipuncture technique. Thereafter, PBMCs were isolated by Ficoll-Hypaque density gradient method followed by RNA extraction. A total of 1 μg of RNA was used to make complementary DNA for RT-PCR.

### PBMC Isolation

Peripheral blood monocytes were isolated from EDTA containing whole blood using histopaque-1077 by recommended protocol.^14^ Total RNA was extracted from the isolated cells by mini RNA extraction kit (Gene-aid, Taiwan).

### Preparation of cDNA

Extracted RNA was reverse transcribed with 1 μl of MMuLV reverse transcriptase in 5 μl volume of 5 × RT buffer (250 mMTris-HCl pH 8.3, 375 mMKCl, 15 mM MgCl_2_) supplemented with dNTPs and 1 μl of oligo (dT).

### Polymerase Chain Reaction

An aliquot of 4 μl of the cDNA was used for polyme-rase chain reaction (PCR) amplification in 2.5 μl of 10X buffer solution (100 mMTris-HCl pH 9.3, 500 mMKCl, 1% Triton X-100) containing 0.5 μl dNTPs, primers (0.5 μl each) and 0.2 μl of Taq DNA polymerase. PECAM-1 and house keeping cDNA fragments were amplified by 45 cycles (94°C—1 minute, 48°C-58°C—45 seconds and 72°C—30 seconds per step) with an initial denaturation at 95°C for 4 minutes using forward and reverse primers F-CAACGAGAAAATGTCAGA; R-GGAGCCTTCGTTCT-AGAGT and F-CCAGCTCACCATGGATGATG; R-ATGCCGGAGCCGTTGTC generating amplicons of 259 bp and 56 bp respectively.

Each amplified product was subjected to agarose gel , electrophoresis (100 V, 45 minutes) along with a 1,000 bp DNA ladder. The electrophoretic bands were visualized by an attached camera in a gel documentation system by using UV transilluminator (Wealtec Dolphin View, Nashville, TN, USA). DNA fragments of selected host genes were detected and photographed. The integrated density of DNA band was evaluated by using the software Image J 1.42 (Broken Symmetry Software, Naperville, IL, USA) and further normalized against the housekeeping gene beta actin used as internal control to define the expression of respective genes by the density of the band.

## STATISTICAL ANALYSES

All necessary information and clinical data was systematically recorded in a predesigned data collection sheet. Results were expressed as mean ± standard deviation (SD) or percentage. Statistical analysis of HBV DNA value was performed after log_10_ conversion. The statistical significance of intergroup differences was evaluated by means of Mann-Whitney U-tests. Spearman correlation was performed for correlation analysis. Statistical analysis was made using SPSS 17.0 software. p-value of <0.05 considered as statistically significant.

## RESULTS

### Samples

All the study subjects were adult in all three analyzed groups (untreated and treated CHB patients and healthy controls) with the mean age 31.20 ± 6.03, 29.50 ± 6.13 and 32.90 ± 4.01 years with the male to female ratio were 1.3:1, 1.14:1 and 1:1 respectively (Table 1).

### Expression of PECAM-1 Gene in Study Subjects

**Fig. 1:**
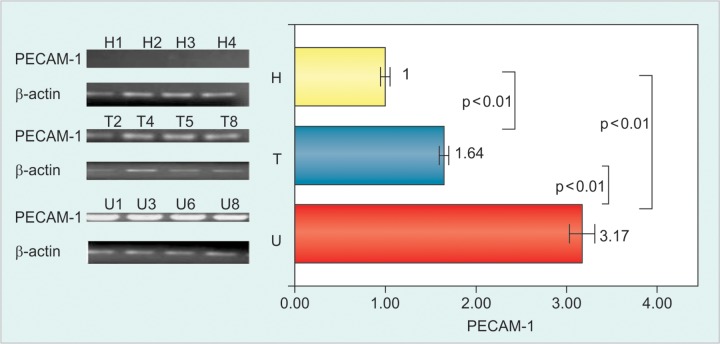
Expression of PECAM-1 mRNA in PBMCs from untreated (U), treated (T) CHB patients and healthy (H) control. The expression of PECAM-1 was detected by RT-PCR and gel intensity was measured by image J 1.42 software. Normalization of the result was done by the intensity of housekeeping gene p-actin (Bar-SD). Data are shown as mean ± SE. Mann-Whitney U-test was done (p < 0.05) indicates statistical significance

**Graph 1:**
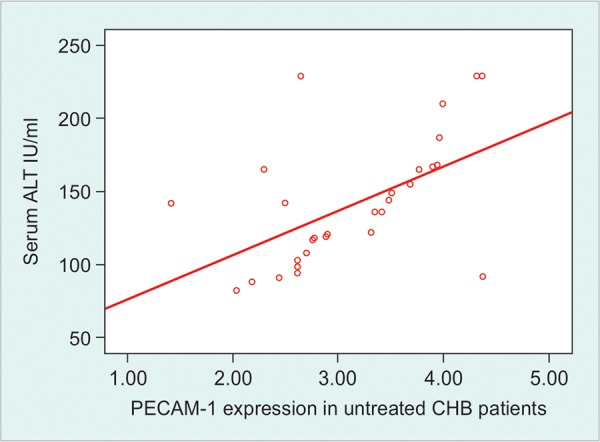
Relationship between expression of PECAM-1 mRNA and serum ALT levels among untreated CHB patients. Spearman correction text was done

**Graph 2:**
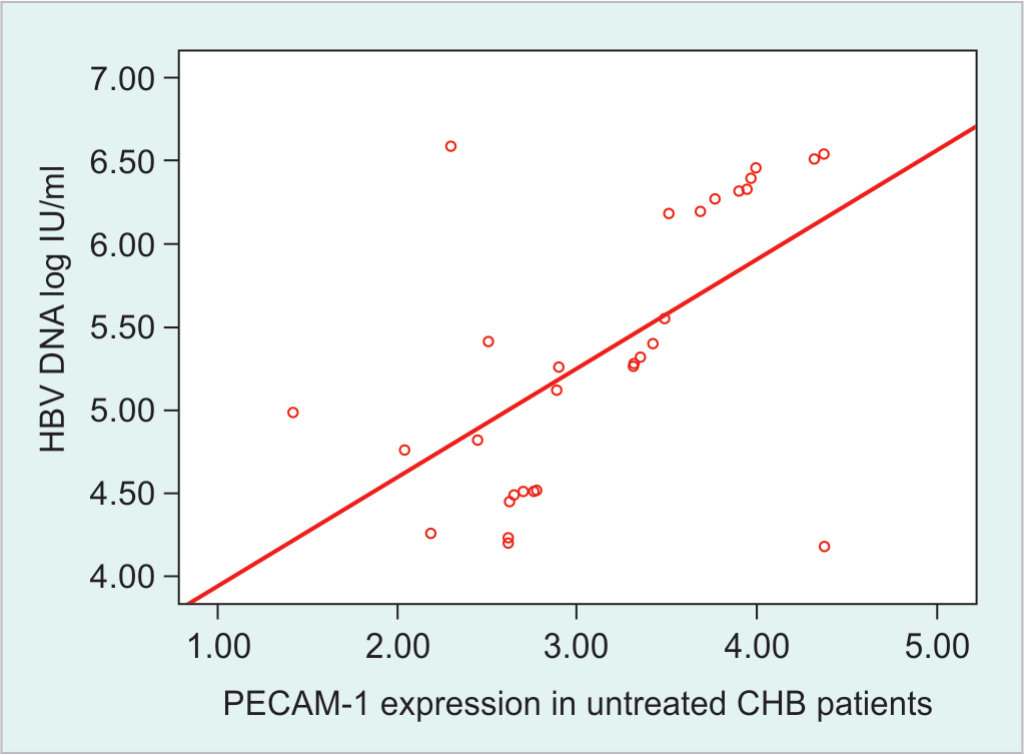
Relationship between expression of PECAM-1 mRNA and serum HBV DNA load among untreated CHB patients. Spearman correction text was done

The mean normalized values of expression of PECAM-1 gene were 3.17 ± 0.75, 1.64 ± 0.29 and 1.0 ± 0.13 fold among untreated, treated CHB patients and healthy control. The expression was significantly higher in untreated than treated CHB patients and healthy control (p < 0.01). The expression was also higher in treated CHB patients than the healthy control (p < 0.01). This gene expression had moderately positive correlation with serum ALT levels (r = 0.580, p < 0.01) as well as HBV DNA load (r = 0.545, p < 0.05) among untreated CHB patients. In treated patients there was moderately positive correlation of this gene with serum ALT levels (r = 0.566, p < 0.01) and HBV DNA load (r = 0.591, p < 0.01) (Fig. 1, Graphs 1 and 2).

## DISCUSSION

HBV infection leads to inflammatory processes of different grades that involve activation of adhesion molecules and cytokines, which facilitate recruitment of leukocytes to inflammatory areas. The evidence supporting this suggestion is the increase in expression of PECAM-1 in PBMCs of CHB patients as observed from the performed study.

**Table 1:** **Table 1:** Comparison of parameters among untreated and treated CHB patients and healthy control

*Parameter*		*Uotreated CHB* * (n = 30)*		*Treated CHB* * (n = 30)*		p*		*Healthy cnotrnl* * (n = 10)*		p**		p***	
Age (year) mean (SD)		31.20 ±6.03		29.50 ± 6.13		NS		32.90 ±4.01		NS		NS	
Sex: (M/F)		1.3:1		1.14:1		NS		1:1		NS		NS	
ALT (U/L) mean ± SD		138.37 ±47.67		40.63 ±3.06		<0.05		29.10 ±2.96		<0.05		<0.05	

p*, according to Mann-Whitney U-test for quantitative data and chi-square test for qualitative data; p* values treated *vs* untreated CHB patients; p**, untreated CHB *vs* healthy control; p***, treated CHB patients *vs* healthy control; M: Male; F: Female; NS: Not significant; SD: Standard deviation; p < 0.05 indicates statistically significant

Significantly higher expression of PECAM-1 mRNA in untreated than treated CHB patients suggests that PECAM-1 may reflect that its expression can be induced by an active inflammatory process. Antiviral therapy minimizes extent of inflammatory process and therefore contributes to down regulation of PECAM-1 gene expression. This finding is consistent with our present study where lower expression of PECAM-1 gene was observed in treated CHB patients.

**Graph 3:**
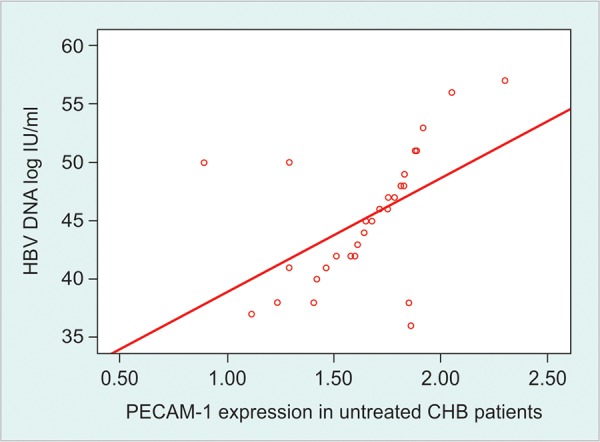
Relationship between expression of PECAM-1 mRNA and serum ALT levels among treated CHB patients. Spearman correlation text was done

Correlation between PECAM-1 expression and aminotransferase activity a marker of inflammatory process also supports this suggestion. Moreover, in this study the lower expression in treated patients with normal ALT levels were still significantly higher than in healthy subjects, which indicate that PECAM-1 also points to the ongoing inflammatory process (Graph 3).

There is no data available with respect to the relationship between PECAM-1 gene expression and inflammatory activity in CHB. However, in the case of cirrhosis weak immunoreactivity for PECAM-1 observed on sinusoidal epithelial cells (SEC) and endothelial cells of vessels in portal tracts in normal liver increases significantly.^15^

**Graph 4:**
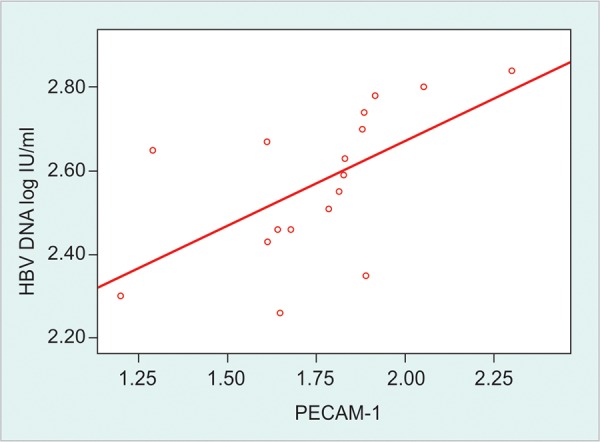
Relationship between expression of PECAM-1 mRNA and HBV DNA load among treated CHB patients. Spearman correlation text was done. Treated patients with undetectable HBV DNA were not included in this correction

Studies have reported that the level of HBV DNA in serum is an important indicator to evaluate viral replication in the host and the clinical therapeutic effects.^16^ In the present study, there was positive correlation between PECAM-1 gene expression and HBV DNA load. This is due to the fact that weak virus specific T cell response fails to clear the virus rather causing inflammatory activity (Graph 4).

It is thought that PECAM-1, a signal transducer in cells, may be responsible for upregulation of integrins expression, their binding to other molecules required for transmigration of inflammatory cells into tissues.^17^ Thus, during an inflammatory process inflammatory cells recruit into the concerned tissue resulting tissue damage.

Infection with HBV results in upregulation of PECAM-1 mRNA in PBMCs. Our findings are consistent with the importance of PECAM-1 in CHB pathogenesis and suggest that it is related to inflammatory activity as assessed by serum ALT levels. Therefore, detection of PECAM-1 may be an additional feasible marker of inflammatory activity. Our analysis has shown that PECAM-1 expression measurement was useful in distinguishing untreated and treated CHB patients and healthy controls. Antiviral therapy, by reducing the inflammatory activity, decreases markedly expression of PECAM-1. Moreover, positive correlation between HBV DNA and this gene expression suggests aberrant and weak immune response in CHB patients. Thus, PECAM-1 may be used as marker for assessment of therapeutic response along with other markers, like serum ALT levels, viral load and HBeAg status. Further studies on a greater number of patients are necessary for better determination of the role of PECAM-1 in the pathogenesis, diagnosis and management of CHB.
